# Congenital stomised omphalocele: a case report and scoping literature review

**DOI:** 10.3389/fped.2026.1832797

**Published:** 2026-06-10

**Authors:** Giada Loria, Alessandra Fichera, Pasqua Betta, Roberta Rocca, Vincenzo Di Benedetto, Maria Grazia Scuderi

**Affiliations:** 1 Department of Medical and Surgical Sciences and Advanced Technologies “G.F. Ingrassia”, Unit of Pediatric Surgery, Policlinico G. Rodolico – San Marco, University of Catania, Catania, Italy; 2 Neonatal Intensive Care Unit, Policlinico G. Rodolico – San Marco, Catania, Italy

**Keywords:** abdominal wall defect, congenital stoma, fistula, neonatal surgery, omphalocele

## Abstract

**Background:**

Omphalocele is a congenital midline abdominal wall defect, rarely associated with a congenital stoma. This unusual presentation presents unique diagnostic and surgical challenges, with only a handful of cases reported in the literature.

**Case report:**

We report a male neonate born at 36 + 6 weeks of gestation with a small-to-moderate omphalocele (6 cm) containing an externalized ileal segment that functioned as a congenital stoma. Preoperative evaluation, including contrast study via the stoma, echocardiography, and genetic testing, revealed no associated malformations. During the first month of life, conservative management was adopted, and the stoma remained functional. Subsequently, the infant developed signs of bowel obstruction with persistent bilious vomiting and therefore underwent surgical intervention. Intraoperative findings warranted resection of the affected bowel segment, followed by primary end-to-end anastomosis and direct abdominal wall closure. Histopathologic analysis confirmed congenital spontaneous fistulization without evidence of vitelline duct remnants or heterotopia. The postoperative course was uneventful, with early return to full enteral feeding and normal growth documented at 12-month follow-up.

**Methods:**

A scoping review of PubMed and Scopus identified previously reported cases of omphalocele with congenital stoma. Among 1,428 studies screened, only one met the inclusion criteria.

**Discussion and conclusion:**

Unlike previously reported cases, our patient had no intestinal atresia, suggesting a distinct embryologic mechanism. Omphalocele associated with a congenital stoma is exceptionally rare but can be safely repaired through deferred single-stage intervention in selected cases. Reporting additional cases and fostering multicenter collaboration are essential to improve understanding and guide management.

## Introduction

Omphalocele is a congenital midline abdominal wall defect, whose incidence varies across countries, with an esteem of 1.92 per 10,000 live births in United States (1). It is characterized by herniation of abdominal viscera through the umbilical ring, enclosed by peritoneum and amniotic sac (2). A key differential diagnosis is gastroschisis, which presents as a paraumbilical full-thickness abdominal wall defect, usually right-sided, with uncovered bowel directly exposed to amniotic fluid, leading to inflammatory serositis (3).

Small omphaloceles [<5 cm] typically contain only small bowel, whereas giant omphaloceles may include the liver and are frequently associated with pulmonary hypoplasia and viscero-abdominal disproportion, often requiring staged repair (4). Omphalocele is often associated with cardiac malformations, chromosomal abnormalities, and syndromic presentations (5, 6).

A rare and atypical presentation involves the presence of an externalized, mucosa-lined bowel end visible at birth on the surface of omphalocele membrane, effectively functioning as a congenital enterostomy. From an epidemiological standpoint, stomised omphaloceles are not recognized as a distinct nosological entity in major classification systems, and their true incidence is unknown (7).

Male predominance and maternal age extremes (<20 years or ≥35 years) have been noted as background risk factors for omphalocele in general and likely extend to this rare variant (2). Highly specialized centers and multidisciplinary teams are necessary for neonatal surgical diseases, which are a critical component of pediatric healthcare (8) especially when it represents one clinical “exception” to an already rare disease.

Given the exceptional rarity of omphalocele associated with a congenital stoma, conducting a traditional systematic review with meta-analysis is not feasible, as the available literature lacks sufficient high-quality and homogeneous data for quantitative synthesis. In this context, a scoping review represents the most appropriate methodological approach, as it enables a comprehensive mapping of the existing evidence across diverse aspects, This approach enables mapping of existing evidence on presentation, surgical strategies, outcomes, and research gaps.

## Case presentation

We report the case of a first-born male infant, delivered at 36 + 6 weeks of gestation from a heterologous-assisted pregnancy. Prenatal ultrasound revealed a small-to-moderate omphalocele measuring approximately 6 cm and containing intestinal loops, without evidence of associated anomalies. The pregnancy was monitored at a tertiary care centre. Delivery was performed by elective caesarean section due to maternal abdominal pain and breech presentation. Apgar scores were 9 and 10 at 1 and 5 min, respectively. Birth weight was 3,000 g, with adequate extrauterine adaptation.

**Figure 1:**
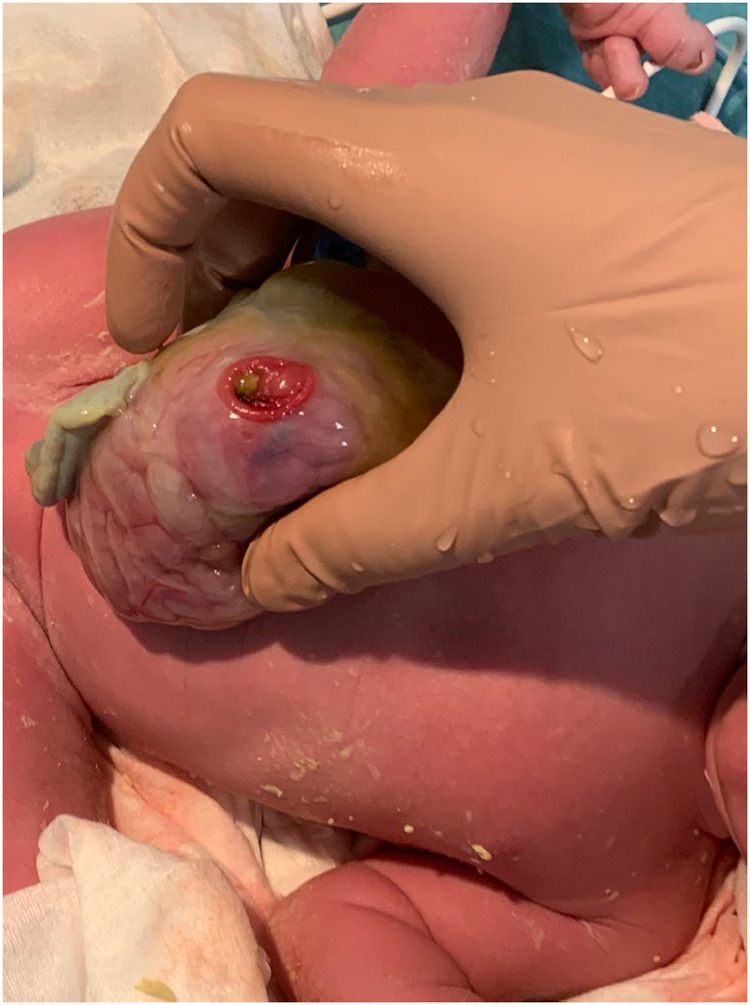
Preoperative view showing a 6 cm omphalocele with partial membrane ulceration and a congenital ileal stoma expelling meconium.

Postnatal examination confirmed a 6 cm omphalocele with partial sac ulceration and a mucosa-lined bowel segment spontaneously draining meconium, consistent with a congenital enterostomy (Figure 1). The remainder of the examination was normal.

Diagnostic investigations were performed as part of the malformation work-up, given the high incidence of associated anomalies in omphalocele, and to guide surgical management. Abdominal x-ray demonstrated intestinal loops within the sac without free air or pathological air–fluid levels. A contrast study via the stoma revealed a patent colon with microcolon pattern due to disuse, opacification of the terminal ileum beyond the ileocecal valve, and no evidence of malrotation, obstruction, or fistulas. These findings were crucial to exclude associated intestinal atresias, which would have significantly altered the surgical approach. Echocardiography revealed a structurally normal heart with a patent foramen ovale and mild left-to-right shunt, with evidence of spontaneous closure of the ductus arteriosus at the follow-up with echocardiography after about 90 h from birth. Cranial ultrasound demonstrated normal midline structures, non-dilated ventricles, and an intact corpus callosum. Genetic analysis confirmed a 46, XY karyotype, with no dysmorphic features or associated anomalies.

For the first month of life the patient was asymptomatic, the stoma was regularly functional. At 34th day of life, the infant developed signs of bowel obstruction with persistent bilious vomiting. Conservative treatment with bowel rest and nasogastric tube placement have been initially tried, without improvement of clinical condition, so the multidisciplinary team decided for surgical intervention.

Exploratory laparotomy revealed a congenital ileal stoma approximately 35 cm proximal to the ileocecal valve. The affected segment was resected, and a primary end-to-end anastomosis performed. Direct abdominal wall closure was achieved with acceptable intra-abdominal pressure. The infant required brief mechanical ventilation, was extubated on day 2, and commenced enteral feeding on day 3. Full feeding was achieved gradually with preserved bowel function.

Post-operative course was complicated by the onset of fever associated to the elevation of inflammatory markers. On blood culture, *Malassezia furfur* have been isolated, requiring temporary antifungal therapy. No hemodynamic instability has been observed. According to the Clavien–Madadi classification (9), these corresponded to grade I–II complications, with no major adverse events observed.

Histopathological analysis substantiated the diagnosis of a congenital spontaneous fistulization of the bowel to the omphalocele membrane, with no histological evidence of heterotopia, vitelline duct remnants nor other signs suggestive of a Meckel's diverticulum.

**Figure 2:**
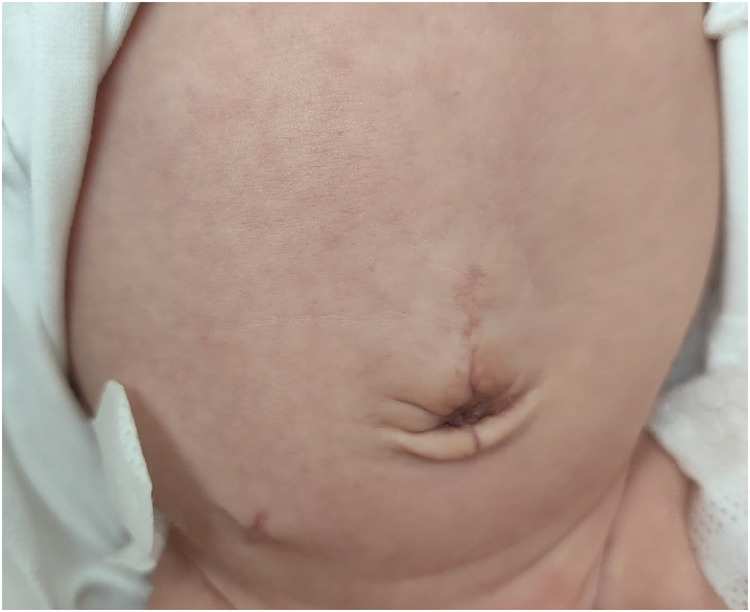
Postoperative aspect at follow-up.

Periodic follow-up up to 12 months showed steady weight gain, normal bowel function, absence of surgical complications and an excellent esthetic result (Figure 2).

## Materials and methods

A male neonate with omphalocele and congenital ileal stoma was treated at our Pediatric Surgery Unit. Clinical details, diagnostic investigations, surgical management, and follow-up were reviewed. To map the literature concerning with an intestine opening to the surface of an intact omphaloceles sac, a systematic scoping literature review was conducted using the PubMed and Scopus databases.

The search strings used included various combinations of the following keywords: omphalocele, exomphalos, stoma, ostomy, ileostomy, colostomy, fistulization, fistula.

No restrictions were applied regarding the publication year. Articles were filtered by language, and those not published in English, French, or Italian were excluded.

The inclusion criteria encompassed studies reporting data on paediatric patients diagnosed with a congenital abdominal wall defect and presenting a congenital bowel fistulization to the skin or bowel-covering membrane. Studies were considered eligible if they reported information on patient history, clinical presentation, associated malformations, surgical management, postoperative outcomes, and follow-up. Exclusion criteria were applied to studies focusing on patients whose primary diagnosis was other than omphalocele, as well as to all cases in which the external fistulization was considered to originate from vitelline duct anomalies.

Studies were deemed eligible if they reported data on patient history, clinical presentation, associated malformations, surgical management, postoperative outcomes, and follow-up.

Two reviewers independently evaluated each article, and no disagreements were encountered. Full-text articles meeting the selection criteria were reviewed in detail, and study data were extracted in a standardized manner.

The PRISMA-ScR checklist (10) was used to ensure the relevance and completeness of the review.

## Results

In order to map the existing literature and compare it with our experience, a comprehensive scoping review was conducted using the Scopus and PubMed databases, with additional checks of relevant reference lists and no restrictions on publication year, following the PRISMA guidelines (10).

A total of 1,428 papers were identified from Scopus and PubMed. Of these, 499 were duplicates, and 830 were excluded because they focused on primary diagnoses other than omphalocele. Among the 99 remaining papers, only 1 record (11) met the eligibility criteria.

Etensel et al. (11) provided a detailed intraoperative description, reporting a premature infant with a small omphalocele (22 mm) containing a congenital ileostomy associated with two distal type I ileal atresias. Early repair was successful, consisting of resection of the affected segment, primary anastomosis, and abdominal wall closure.

## Discussion

Omphalocele itself likely reflects a complex underlying embryological disturbance (12). When it is associated with a congenital stoma represents an extremely rare congenital anomaly, with very few cases reported in the literature (7).

When multiple congenital anomalies exist, significant diagnostic and surgical challenges are posed, particularly in defining the continuity and viability of the gastrointestinal tract at birth (13).

Our patient represents an isolated spontaneous fistulization without atresia, differing from previously reported cases and suggesting a distinct pathogenesis.

The presence of the congenital stoma allowed immediate recognition of bowel discontinuity and facilitated targeted contrast imaging to confirm colonic patency and define distal anatomy. Deferred single-stage repair was feasible, safe, and yielded excellent outcomes both clinical and esthetic, which is nowadays considered the main objectives is to achieve In pediatric surgery (8). Perioperative priorities include fluid balance, mucosal protection, and infection control. In the absence of major malformations, prognosis parallels that of isolated omphalocele, with mortality primarily linked to cardiac or chromosomal anomalies.

Congenital spontaneous fistulization of the bowel to the omphalocele membrane was diagnosed as confirmed by histopathological analysis, and there was no sign of heterotopia, vitelline duct remnants, or other symptoms that would indicate an absence of a Meckel's diverticulum, which was the team's first diagnostic hypothesis and the reason to perform a literature review.

Our scoping review identified only one comparable case reported by Etensel et al. (11), describing a minor omphalocele with a congenital stoma associated with multiple ileal atresias, which was successfully managed with resection and primary anastomosis. By contrast, our patient had no additional intestinal anomalies beyond the congenital stoma, leaving the underlying etiology unclear and making it difficult to propose a definitive explanation.

Recently, McNickle et al. (7) reported a series of exomphalos with intestinal fistulation, analysing clinical features, management strategies, and embryo-pathogenesis. Similar to these reports, our patient presented with a small-to-moderate omphalocele containing a spontaneously externalized bowel segment, consistent with a congenital enterostomy. Early surgical intervention allowed resection of the affected segment, primary anastomosis, and abdominal wall closure, leading to an excellent outcome.

In the reported cases, the congenital ostomy on the omphalocele membrane was attributed to a persistent vitelline duct or Meckel's diverticulum. These cases were therefore considered unsuitable for inclusion in our review, as histopathological analysis of our patient excluded heterotopia or vitelline duct remnants, supporting the diagnosis of spontaneous congenital fistulization and suggesting a different etiology of the defect.

The present study shares the same limitations as the available literature: the infinitesimal number of reported patients, the retrospective nature of published data, and the absence of standardized treatment protocols. Since no standardized treatment strategy is currently available for congenital stomised omphalocele, we proposed a repair which took into account a deep preoperative evaluation, including echocardiography, genetic testing and a contrast study of bowel anatomy and viability prior to surgery.

A conservative approach was initially adopted, and surgery was intentionally deferred as long as possible, until the onset of clinical signs, such as persistent bilious vomiting, indicating the need for operative management.

Given the extreme rarity and absence of standardized guidelines, management should be individualized according to defect size, bowel viability, and associated anomalies. By integrating systematic assessment, careful protection of exposed tissues, targeted imaging, and a tailored surgical strategy, we aimed to offer a reproducible management pathway that could be adapted to other rare and complex presentations of stomised omphalocele.

## Conclusion

Omphalocele associated with a congenital stoma is an exceptionally rare anomaly. This case demonstrates that spontaneous congenital fistulization of bowel to the omphalocele membrane may occur independently of vitelline duct remnants. We propose an approach where surgical intervention has been deferred until the appearance of clinical signs or symptoms indicating the need for operative management, allowing to rule out the presence of associated malformation. Single-stage resection and closure are safe in selected patients, yielding excellent outcomes. Continued case reporting and multicenter collaboration are essential to refine diagnostic understanding, surgical management, and prognostic counseling.
